# Does a Transition to Single-Occupancy Patient Rooms Affect the Incidence and Outcome of In-Hospital Cardiac Arrests?

**DOI:** 10.1177/19375867241226600

**Published:** 2024-02-23

**Authors:** Ralph Pruijsten, Gerrie Prins-van Gilst, Chantal Schuiling, Monique van Dijk, Marc Schluep

**Affiliations:** 1 Section Nursing Science, Department of Internal Medicine, Erasmus University Medical Center, Rotterdam, the Netherlands; 2 Department of Intensive Care, Ikazia Hospital, Rotterdam, the Netherlands; 3 Department of Intensive Care, Erasmus University Medical Center, Rotterdam, the Netherlands; 4 Department of Anesthesiology, Erasmus University Medical Center, Rotterdam, the Netherlands; 5 Department of Anesthesiology and Intensive Care, Bravis Hospital, Bergen op Zoom, the Netherlands

**Keywords:** single-occupancy rooms, hospital design, cardiac arrest, cardio pulmonary resuscitation, advanced cardiac life support

## Abstract

**Background:**

It is proposed that patients in single-occupancy patient rooms (SPRs) carry a risk of less surveillance by nursing and medical staff and that resuscitation teams need longer to arrive in case of in-hospital cardiac arrest (IHCA). Higher incidences of IHCA and worse outcomes after cardiopulmonary resuscitation (CPR) may be the result.

**Objectives:**

Our study examines whether there is a difference in incidence and outcomes of IHCA before and after the transition from a hospital with multibedded rooms to solely SPRs.

**Methods:**

In this prospective observational study in a Dutch university hospital, as a part of the Resuscitation Outcomes in the Netherlands study, we reviewed all cases of IHCA on general adult wards in a period of 16.5 months before to 16.5 months after the transition to SPRs.

**Results:**

During the study period, 102 CPR attempts were performed: 51 in the former hospital and 51 in the new hospital. Median time between last-seen-well and start basic life support did not differ significantly, nor did median time to arrival of the CPR team. Survival rates to hospital discharge were 30.0% versus 29.4% of resuscitated patients (*p* = 1.00), with comparable neurological outcomes: 86.7% of discharged patients in the new hospital had Cerebral Performance Category 1 (good cerebral performance) versus 46.7% in the former hospital (*p* = .067). When corrected for telemetry monitoring, these differences were still nonsignificant.

**Conclusions:**

The transition to a 100% SPR hospital had no negative impact on incidence, survival rates, and neurological outcomes of IHCAs on general adult wards.

## Introduction

An in-hospital cardiac arrest (IHCA) is the most severe adverse event for hospitalized patients. A systematic review described 1–6 IHCA events per 1,000 hospital admissions (Schluep et al., 2018). Cardiopulmonary resuscitation (CPR), aimed to restore circulation, leads to survival-to-discharge rates of 15.0% and 17.6% (Schluep et al., 2018; Zhu et al., 2016). Previous studies have demonstrated that outcomes are affected by nonmodifiable patient characteristics like age, gender, and preexisting conditions, and modifiable event characteristics like the location of the IHCA (Andersen et al., 2019; Piscator et al., 2019). The current European Resuscitation Council (ERC) guidelines emphasize the important role of early recognition of a circulatory arrest within the resuscitation “chain-of-survival,” because this is associated with improved outcomes (Kronick et al., 2015).

A potential threat for early recognition of an IHCA is the transition from multibedded patient rooms to single-occupancy patient rooms (SPRs). Worldwide, more and more hospitals choose to transition to 100% SPRs (Maben et al., 2015). Benefits of this trend include improved patient well-being and privacy and better infection control (Cusack et al., 2023; Nash et al., 2021; Schreuder et al., 2016; Ulrich et al., 2006). However, disadvantages have also been described, such as feelings of isolation and reduced social interactions of patients (Taylor et al., 2018). In addition, Søndergaard and colleagues showed in a recent scoping review that nurses report longer walking distances and stress of worrying about missing data, jeopardizing patient safety (Søndergaard et al., 2022). However, little is known about the relationship between SPRs and important clinical outcomes such as IHCAs.

In May 2018, our university medical center, located in Rotterdam, the Netherlands, relocated to a newly built hospital with 100% SPRs. To address concerns raised by our own staff, as well as a lack of evidence pertaining to this subject, we decided to conduct the current study. The aim of this study was to see whether the incidence and outcomes of IHCAs in the new setting with solely SPRs differed from that in the one with mainly multibedded patient rooms.

***The aim of this study was to see whether the incidence and outcomes of IHCAs in the new setting with solely SPRs differed from that in the one with mainly multibedded patient rooms.***

## Method

### Design

Our study is a prospective before–after study in a large university hospital (1,125 beds) embedded in two larger studies: one evaluating the effects of the transition from a hospital with mainly multibedded rooms to solely SPRs on nurses and patients (WELCOME; approval of this study was obtained from the Medical Ethics Committee of Erasmus University Medical Center Rotterdam, the Netherlands [MEC-2017-1103]) and one examining outcomes after in-hospital resuscitation (ROUTINE; Schluep et al., 2021). The ROUTINE study was considered subject to the ‘Wet medisch-wetenschappelijk onderzoek met mensen’ (meaning ‘Dutch Medical Research Involving Human Subjects Act’) and was approved by the Erasmus University Medical Centre Medical Ethics Committee (MEC 2016-563/NL-55661.078.16).

Data of the former hospital were collected from January 1, 2017 through May 18, 2018 (16.5 months). The relocation to the new hospital took place on May 18, 2018. Data of the new hospital were collected from May 19, 2018 through September 9, 2019 (16.5 months).

### Setting and Patients

All general adult nursing wards of our hospital were involved in this study. In the former hospital, each of these wards held around 32 beds and mainly contained two-person and four-person rooms. In some circumstances, a two-person room was used as a single-patient room. The wards in the new hospital each have around 24–32 SPRs. Intensive care units, operating rooms, cardiac catheterization laboratory, and the emergency department were excluded because they provide continuous monitoring of all vital signs, and the staff was already accustomed to single rooms in the old setting. We excluded patients who had already been resuscitated outside the hospital within 24 hr prior to a new cardiac arrest and children aged 16 or younger. The usage of telemetry monitoring (three-lead electrocardiogram (ECG)), in which patients continuously carry a telemetry box so they can freely mobilize on the ward, was not an exclusion criterion. The reason for this was that this category of patients had also relocated from multibedded rooms to SPRs. To be able to compare groups with and without telemetry monitoring, we registered whether or not patients carried this monitoring.

In case of a IHCA, medical and nursing staff started basic life support (BLS) and immediately alerted the CPR team. This team started advanced life support (ALS). All healthcare professionals of our hospital are trained to provide BLS, to the standards of the ERC.

### Outcome Measures

Primary outcome measure was the incidence of resuscitations, secondary outcome measures were return of spontaneous circulation, cause of cardiac arrest, delay in start BLS and lead time of the CPR team (delay in start ALS), survival to discharge, and Cerebral Performance Category (CPC) score at discharge.

### Data Collection

All patients were prospectively included through registrations done by the in-house CPR-team, and registrations were crosschecked with ICU-admissions for cardiac arrest. For both quality registration, as financial administration purposes, all CPR attempts are registered as anesthesiology consultations. All data were extracted from the Electronic Patient File and entered into OpenClinica system for trial registration. In-hospital follow-up was done by the main investigator (M.S.) until hospital discharge. After discharge, survival was checked with the Dutch Personal Records Database (BRP). CPR was defined as starting chest compressions in the absence of signs of circulation. Delay to BLS was defined as the time between last-seen-well and start of CPR. For each cardiac arrest, it was documented whether it was witnessed or not. The arrest was considered witnessed if the loss of consciousness and the circulatory arrest occurred in the presence of nursing or medical staff. The lead time of the CPR team was documented in the patient file by the nursing staff. The first observed heart rhythm after connection of the defibrillator by the CPR team was also documented. We looked at discharge destination and the CPC score, which provides insight into cognitive functioning, and the Age-adjusted Charlson Comorbidity Index (ACCI), which provides a prognosis of the 1-year survival rate given the comorbidity (Edgren et al., 1994; Quan et al., 2011). Pseudonymized data of the patients and resuscitations were collected from the patient files and stored on a secure server of the hospital.

### Data Analysis

The data were analyzed using IBM Statistical Package for the Social Sciences 28 (BM SPSS Statistics for Windows, Version 28.0, Armonk, NY: IBM Corp). To compare the data of former and new hospital, a χ^2^ test or Fisher exact test was used as appropriate for categorical variables and a t test or Mann–Whitney U test for continuous variables. Statistical significance was defined as *p* < .05.

## Results

### Patient Characteristics

A total of 102 resuscitations were performed on general adult patient wards during the study period. It concerned 51 in the former hospital (with mainly multibedded patient rooms) and 51 in the new hospital (with solely SPRs). An overview of patient characteristics is given in Table 1. Mostly men suffered cardiac arrests: 68.6% in the former hospital and 68.6% in the new hospital (*p* = 1.000). No significant differences in patient age, Age-adjusted Charlson Comorbidity Index (ACCI), body mass index (BMI), and proportion of smokers between the two groups were found. Both before and after the transition to the new hospital, the majority of resuscitated patients were admitted to surgical wards.

**Table 1.** Patient Characteristics of All Resuscitations on Patient Wards in Former and New Hospital.

Patient Characteristics	Former Hospital, *N* = 51	New Hospital, *N* = 51	Total, *N* = 102	*p* Value
Sex: man, *n* (%)	35 (68.6)	35 (68.6)	70 (68.6)	1.000
Age: years, mean (*SD*)	64.4 (10.9)	65.1 (14.0)	64.8 (12.5)	.770
Age-adjusted Charlson Comorbidity Index, median (IQR)	4 (3–6)	5 (3–6)	4 (3–6)	.264
Body mass index, mean (*SD*)	26.5 (3.9)	26.1 (4.8)^a^	26.3 (4.4)	.605
Smoking, *n* (%)				.597
Yes	7 (13.7)	12 (23.5)	19 (18.6)	
Quit	18 (35.3)	14 (27.5)	32 (31.4)	
No	20 (39.2)	18 (35.3)	38 (37.3)	
Unknown	6 (11.8)	7 (13.7)	13 (12.7)	
Admission specialty, *n* (%)				.358
Cardiology	6 (11.8)	9 (17.6)	15 (14.7)	
Surgery	12 (23.5)	15 (29.4)	27 (26.5)	
Internal medicine	8 (15.7)	8 (15.7)	16 (15.7)	
Cardiothoracic surgery	7 (13.7)	11 (21.6)	18 (17.6)	
Pulmonary medicine	8 (15.7)	2 (3.9)	10 (9.8)	
Neurology	4 (7.8)	1 (2.0)	5 (4.9)	
Neurosurgery	2 (3.9)	1 (2.0)	3 (2.9)	
Gastroenterology and hepatology	2 (3.9)	1 (2.0)	3 (2.9)	
Ear, nose, and throat surgeries	0	2 (3.9)	2 (2.0)	
Urology	1 (2.0)	1 (2.0)	2 (2.0)	
Gynecology	1 (2.0)	0	1 (1.0)	

*Note*. IQR = interquartile range

^a^ Two missing values (4%).

Of the 27 resuscitated patients with admission specialty surgery, eight patients (29.6%) had undergone surgery less than 72 hr ago. The other patients had undergone surgery longer ago or were not postoperative. There was one surgical emergency (necrotizing fasciitis) and no trauma emergencies. Of the 18 resuscitated patients with admission specialty cardiothoracic surgery, five patients (27.8%) had undergone surgery less than 72 hr ago. There were three cardiothoracic surgical emergencies: two patients with type A aortic dissection and one patient with cardiac tamponade.

### Characteristics of the IHCA and Outcomes

Table 2 shows the characteristics of the cardiac arrests and their outcomes after resuscitation in the former versus the new hospital. The majority of the arrests in both groups were witnessed: 74.5% versus 78.4% (*p* = .816). A majority of these were witnessed by the nursing staff (60 of 78: 76.9%; 29 in the former and 31 in the new hospital). The other arrests were witnessed by physicians, visitors, or fellow patients. First observed heart rhythm established at rhythm check did not differ significantly before and after relocation. Most frequent first-registered heart rhythm in both groups was pulseless electric activity (PEA). Of the 57 patients with a PEA arrest, the most common cause was hypoxia (21 patients, 36.8% of PEA arrests), mostly based on (aspiration) pneumonia or mucus stasis. Other common causes were sepsis with multiorgan failure (eight patients, 14.0%), bradycardia/Atrioventricular conduction disorders (seven patients, 12.3%) and pulmonary embolisms (six patients, 10.5%). The proportion of asystole as first observed rhythm did not differ between the two groups (*p* = .654). Median time between last-seen-well and start BLS was 0 min in both groups (*p* = .351). In the former hospital, four resuscitated patients (7.8%) were last seen well more than 10 min before the start of BLS (range 20–50 min). In the new hospital, these were five patients (9.8%; range 15–105 min). The other resuscitated patients (>90%) were conscious and responsive 10 min or shorter before the start of BLS. Most patients were able to alert the nursing staff themselves before the cardiac arrest, or the nursing staff kept an extra eye on the patients because they were clinically deteriorating. In a few cases, the nurses were alerted by visitors or fellow patients. Median time delay until start ALS after starting BLS was 3 versus 4 min (*p* = .212). Return of spontaneous circulation was achieved in more than half of the cases of CPR in both groups, 58.8% versus and 60.8%. In the former hospital, 30.0% of resuscitated patients survived until hospital discharge, versus 29.4% in the new hospital.

**Table 2.** Characteristics of Circulatory Arrests and Outcomes of Resuscitations in Former and New Hospital.

Characteristics of Circulatory Arrests and Outcomes of Resuscitations	Former Hospital, *N* = 51	New Hospital, *N* = 51	Total, *N* = 102	*p* Value
Witnessed arrest, *n* (%)	38 (74.5)	40 (78.4)	78 (76.5)	.816
Telemetry monitoring, *n* (%)	12 (23.5)	16 (31.4)	28 (27.5)	.506
First observed heart rhythm, *n* (%)				.196
Asystole	12 (23.5)	15 (29.4)	27 (26.5)	
Pulseless electrical activity	30 (58.8)	27 (52.9)	57 (55.9)	
Ventricular fibrillation	6 (11.8)	6 (11.8)	12 (11.8)	
Pulseless ventricular tachycardia	3 (5.9)	0	3 (2.9)	
Unknown	0	3 (5.9)	3 (2.9)	
Time between last-seen-well and start basic life support (minutes), median [IQR]	0 [0–3] (range: 0–50)	0 [0–1] (range: 0–105)	0 [0–2] (range: 0–105)	.351
Time to arrival CPR team and start advanced life support (minutes), median [IQR]	3 [2–5] (range: 0–10)	4 [2–5]^a^ (range: 0–10)	3 [2–5] (range: 0–10)	.212
Return of spontaneous circulation (ROSC), yes, *n* (%)	30 (58.8)	31 (60.8)	61 (59.8)	1.000
Survival to discharge, yes, *n* (% of CPRs)	15 (30.0) ^b^	15 (29.4)	30 (29.7)	1.000

*Note*. IQR = interquartile range; CPR = cardiopulmonary resuscitation.

^a^ Three missing values (6%).

^b^ Two of the CPR procedures involved the same patient, during the same hospital admission.

At hospital discharge, the CPC score was 1 (good cerebral performance, no or mild disability) in 46.7% in the former hospital and 86.7% in the new hospital (*p* = .067) as shown in Table 3. The majority of patients could be discharged home: 73.3% versus 66.7%.

**Table 3.** Cognitive State and Destination of Discharged Patients.

Cognitive State and Destination of Discharged Patients	Former Hospital, *N* = 15	New Hospital, *N* = 15	Total, *N* = 30	*p* Value
Cerebral performance category, *n* (%)				.067
1: Good cerebral performance, no or mild disability	7 (46.7)	13 (86.7)	20 (66.7)	
2: Moderate disability, independent in activities of daily living	3 (20.0)	0	3 (10.0)	
3: Severe disability, dependent in activities of daily living	5 (33.3)	2 (13.3)	7 (23.3)	
4: Coma or vegetative state	0	0	0	
Discharge destination, *n* (%)				.295
Home	11 (73.3)	10 (66.7)	21 (70.0)	
Rehabilitation center	1 (6.7)	0	1 (3.3)	
Nursing home	3 (20.0)	2 (13.3)	5 (16.7)	
Other hospital	0	3 (20.0)	3 (10.0)	

We could not obtain exact bed occupancy data during the study period, since the organizational structure of the nursing wards in the new hospital is different from that of the former hospital. We assume that occupancy rates were comparable in both study periods, as the total annual number of inpatient nursing days was approximately the same in 2017–2019. Staff numbers and disciplines were the same in both settings. There was a (balanced) reduction in beds a few days before and after the transition. The incidence rate of resuscitations for the entire hospital (including monitored departments such as critical care units, emergency rooms, and operating rooms) during the study period was 1.5/1,000 admissions in the former hospital and 1.7/1,000 in the new hospital (*p* = .952). Because we could not obtain the exact number of admissions to the patient wards included in our study during the study period, an incidence rate could not be calculated. However, we expect no significant difference with hospital-wide data.

### Telemetry Versus Nontelemetry Monitoring

As shown in Table 2, 23.5% of resuscitated patients in the former hospital and 31.4% in the new hospital were on telemetry (*p* = .506). After excluding the patients on telemetry, no significant differences between former and new hospital were seen for all outcomes.

## Discussion

In this prospective observational study, we found no difference in incidence of IHCA at general adult nursing wards, before and after the transition from a hospital with mainly multibedded patient rooms to one with solely single-patient occupancy rooms.

***In this prospective observational study, we found no difference in incidence of IHCA at general adult nursing wards, before and after the transition from a hospital with mainly multibedded patient rooms to one with solely single-patient occupancy rooms***

There are no studies of the relation between SPRs and the incidence of IHCAs. A recent study of Haschemi et al. comes closest to the situation of SPRs. They investigated the relation between patients who were isolated for infection control precautions and their outcome after an IHCA (Haschemi et al., 2022). Studies on incidence of other adverse events (i.e., fall incidents, infection rates, and delirium), before and after transition to SPRs, show heterogeneous results. Singh et al. found a significant increase in fall incidence, the group of Maben found only a temporary increase in falls and Hussain et al. found no significant difference (Hussain et al., 2023; Maben et al., 2015; Singh et al., 2015). Hussain and colleagues hypothesize that falls might not solely be the consequence of staying in a SPR but caused by intrinsic patient factors (Hussain et al., 2023).

A second important finding in our study was the fact that no difference in first established heart rhythm and survival to discharge was found after moving to solely SPRs. This is of particular interest. Since asystole is the final common path for all types of cardiac arrests, the arrest is likely to have lasted longer when the primary arrest rhythm is asystole, as opposed to PEA, ventricular fibrillation, or pulseless ventricular tachycardia (Parish et al., 2018).

***A second important finding in our study was the fact that no difference in first established heart rhythm and survival to discharge was found after moving to solely SPRs***

A proposed disadvantage of the relocation to 100% SPRs is a delay in early recognition of cardiac arrests in patients on general wards. Proposed reasons are less surveillance of nursing staff, lack of social control by fellow patients, and outstretched architecture resulting in longer walking distances (Maben et al., 2016; Søndergaard et al., 2022; Ulrich et al., 2006). Our findings suggest that this was not the case; times between last-seen-well and start BLS are comparable in former and new hospital, even after excluding patients on telemetry monitoring. We expected that in a setting with solely SPRs, a smaller proportion of the IHCAs would be witnessed by healthcare workers. However, in both situations, a similar majority of more than 70% of IHCAs were witnessed by nursing and/or medical staff. These findings are in contrast with the findings of Haschemi et al. They found a factor 1.5 lower survival rate, as well as worse neurological outcome after an IHCA for isolated patients (Haschemi et al., 2022). As in our study, a large majority of first established heart rhythm after the cardiac arrests were nonshockable (PEA and asystole). However, Haschemi and colleagues describe a higher proportion of nonshockable rhythms in the group of isolated patients than in the group of nonisolated patients. This finding could be an indication of a longer time delay between occurrence of the cardiac arrests and start of BLS in this group, leading to poorer outcomes, as stated by the authors as well. In our study, almost all patients in SPRs were nonisolated patients. Possible disadvantages of isolation such as reduced visibility of the patients for the nursing and medical staffs, fewer visits, and the necessity of protective equipment for the staff, delaying CPR, may have played a lesser role in our situation.

## Strengths and Limitations

A strength of this study is its prospective design. Furthermore, we included patients on several surgical and nonsurgical nursing wards of different medical disciplines, which increases the diversity of our sample. We performed the study in two situations, before and after the relocation of the hospital, where other possible influencing factors on outcomes of IHCA and CPR remained largely unchanged, such as BLS- and ALS-training of the staff, the composition of the CPR teams, protocols, and work processes.

Two limitations of the study need to be addressed. First, it is a single-center study with relatively small numbers of cardiac arrests; this format needs to be repeated in other study sites with higher inclusion rates to confirm our conclusions. Second, the completeness of the information obtained is related to the completeness of the reports of medical and nursing staff present during CPR. For the cardiac arrests that were not witnessed, time of last-seen-well was documented by the medical and/or nursing staff of the ward. In case of incomplete registration, the data were supplemented afterward within 24 hr.

## Conclusions

In our hospital, transition to 100% single-patient occupancy rooms has had no negative impact on the incidence, survival rates, and neurological outcomes of IHCA on general adult wards. The results of this study might contribute to a better understanding of the influence of SPRs in hospitals on patient safety.

***The results of this study might contribute to a better understanding of the influence of SPRs in hospitals on patient safety***

## Implications for Practice

- SPRs on general adult wards do not seem to lead to an increased risk of IHCA.
- Survival rates and neurological outcomes of IHCA on general adult wards seem comparable for SPRs and multibedded rooms.
